# Indirect But Lasting: Linking Indirect Aggression Use Over Childhood to Future Adjustment While Considering Conduct Problems

**DOI:** 10.1002/ab.70079

**Published:** 2026-07-31

**Authors:** Stéphanie Boutin, Émiliane Larrivée, Michèle Déry

**Affiliations:** ^1^ Department of Psychology University of Quebec in Montreal Montreal Canada; ^2^ Department of Psychoeducation University of Sherbrooke Sherbrooke Canada

**Keywords:** adolescent wellbeing, aggression, child behavior, outcomes, problem behaviors

## Abstract

Frequent and recurrent indirect aggressive (IA) behaviors (e.g., spreading false rumors, ignoring) are associated with later detrimental consequences. This high IA use appears to be more frequent among children with conduct problems, making it difficult to determine whether the negative outcomes of IA are related to other conduct problems or whether IA makes a distinct contribution. Therefore, the present study aims to determine whether a high trajectory of IA use from around 8 years of age to around 13 years of age is associated with negative outcomes at around 17 years of age controlling for conduct problems and other confounding variables (age, sex, income). Data for this study are drawn from an ongoing longitudinal project involving 744 children (46.8% girls), including 434 with conduct problems, recruited from eight school boards in Quebec, Canada. IA and conduct problems were measured by a composite score of parents‘ and teachers‘ ratings. Outcomes were measured by self‐reported data on depressive and anxiety symptoms, life satisfaction, substance use, delinquent behaviors, social support network, and school dropout risk. Controlling for conduct problems and other confounding variables, analyses using the manual BCH method in Mplus show that high and stable IA use is associated with more problematic substance use, more delinquent behaviors, and a higher probability to drop out of school. Thus, even if conduct problems account for a large proportion of maladjustment, IA explains a significant amount of unique variance and should thus be considered as an important screening indicator.

Aggressive behaviors are frequently associated with physical and verbal acts. However, they also include a more subtle form of behaviors: indirect aggression^1^ (IA). IA involves ostracizing and slandering behaviors that manipulate peer groups to damage victims‘ reputation or sense of belonging (e.g., gossiping, spreading rumors, revealing secrets, disregarding, excluding; Björkqvist et al. 1992a). While most children occasionally engage in IA, a substantial proportion use it frequently and recurrently (Fite and Pederson 2018). In addition to the considerable harm inflicted on victims (Casper and Card 2017), frequent and persistent IA use places children at risk for adjustment difficulties (Murray‐Close et al. 2016). Moreover, frequent IA use often co‐occurs with conduct problems (CP; Ackermann et al. 2019; Boutin et al. 2021). CP include antisocial behaviors such as physical and verbal aggression, rule breaking, deception, and property destruction (American Psychiatric Association 2022). As childhood CP are well known to have long‐term consequences (e.g., Bevilacqua et al. 2018; Kretschmer et al. 2014; Odgers et al. 2008), it remains unclear whether IA is associated with later adjustment difficulties beyond those already linked to CP. Clarifying the long‐term impact of IA is particularly important for girls who tend to rely more on IA than on physical aggression and are therefore less likely to receive services for aggressive behaviors (Smeets and Roeleveld 2016).

## 1 IA

IA emerges as early as age 3 (Crick et al. 2006). At that point, the behaviors are rather rudimentary but later increase in complexity and frequency given the development of new cognitive and social abilities during childhood (Casas and Bower 2018). Its use intensifies with the growing importance of peer relationships and social status in preadolescence, peaking around ages 9–11 (Boutin et al. 2021) or 13 (Karriker‐Jaffe et al. 2008). Further, person‐centered studies have identified distinct developmental trajectories, showing that while IA use decreases over time for some students, a subgroup engages in it more frequently and persistently (Boutin et al. 2021; Vaillancourt et al. 2007). This persistent subgroup, which can represent up to one‐third of samples, appears at risk for later adjustment difficulties (Cleverley et al. 2012).

## 2 IA and Subsequent Adjustment Difficulties

The long‐term consequences of IA use remain debated. On one hand, the mechanisms linking IA perpetration to later adjustment difficulties are still not fully understood. Some authors highlight individual factors, such as deficits in emotion regulation and interpersonal stress, which may contribute to the development of internalizing symptoms (Murray‐Close et al. 2007; Underwood et al. 2011), whereas others emphasize social processes, such as perceived popularity (see Hensums et al. 2023 for a meta‐analysis). IA can be used to gain social status, and affiliation with popular peers can increase engagement in risky behaviors (e.g., substance use, delinquency, reduced school engagement) that are more common among high‐status adolescents (Schwartz and Gorman 2011). Another possible mechanism involves the association between IA and CP. Previous studies have shown that high levels of IA are more common among children and adolescents presenting CP (Ackermann et al. 2019; Boutin et al. 2021; Keenan et al. 2010). In addition, a longitudinal study following youth from ages 10 to 16 found that IA and CP co‐developed over time (Aizpitarte et al. 2017). Given that CP predict later adjustment difficulties (e.g., Bevilacqua et al. 2018; Odgers et al. 2008), IA may also be associated with such difficulties because both types of behaviors can co‐occur within the same individuals.

On the other hand, although IA use has been associated with later maladjustment in longitudinal studies using community samples (e.g., Atherton et al. 2017; Farrell and Vaillancourt 2023; Herrenkohl et al. 2009; Ji et al. 2025; Kamper and Ostrov 2013; Murray‐Close et al. 2007), low levels of IA are often considered normative during the school years (Murray‐Close et al. 2016; Tackett et al. 2013). IA use can even be associated with positive outcomes for youth (e.g., popularity) (see Heilbron and Prinstein 2008 for a review). Consequently, IA has been conceptualized as both a typical and an atypical developmental behavior. A key issue in this debate concerns the frequency and persistence of IA behaviors. The risk of later negative outcomes may be greater among children who engage in IA frequently and persistently, a possibility that requires person‐centered analyses to be investigated.

Only a handful of studies have examined the association between IA trajectories during childhood and adjustment difficulties in late adolescence. In a large community sample, Cleverley et al. (2012) observed that a high and stable trajectory of IA from childhood to adolescence was associated with more delinquent behaviors at age 18 for both boys and girls, relative to a low IA trajectory, but was not associated with internalizing symptoms. Similarly, using a sample of students recruited from public schools, Ehrenreich et al. (2016) reported that a high trajectory of IA from Grades 3 to 12 was associated with higher levels of rule‐breaking behaviors in Grade 12 for both boys and girls, compared to the lowest IA trajectory. No associations were found between IA trajectories and internalizing symptoms. Both studies included a measure of physical aggression but did not control for its effect when examining the associations between IA trajectories and later outcomes. A third study, which modeled a latent IA trajectory from ages 8 to 11 while controlling for physical aggression in a large community sample, found that higher initial levels of IA predicted delinquency and risky behaviors among both boys and girls, as well as depressive symptoms among girls only, at age 15 (Spieker et al. 2012). Finally, Chen and colleagues (2019) identified a chronic high IA trajectory from ages 10 to 16 in another large community sample. Compared to youth following lower IA trajectories, adolescents in the chronic high trajectory exhibited the highest levels of delinquent behaviors and social rejection, as well as the lowest levels of social acceptance, even after controlling for physical aggression perpetration. No sex differences were observed.

Taken together, these findings suggest that high levels of IA may be associated with later maladjustment. However, apart from Chen et al. (2019), previous studies did not control for the contribution of physical aggression or CP when predicting later outcomes. Consequently, it remains unclear whether the observed outcomes are specifically associated with IA.

## 3 The Current Study

Prior studies examining adjustment difficulties associated with IA trajectories have relied primarily on community samples and have not accounted for CP beyond physical aggression. However, research has shown that high levels of IA are most commonly observed among youth presenting CP (Ackermann et al. 2019; Boutin et al. 2021; Keenan et al. 2010), a broader set of difficulties that includes, but is not limited to, physical aggression. To clarify whether later adjustment difficulties stem from IA specifically or from broader CP, the present study includes children with and without CP, an approach that is novel in IA research. Moreover, although studies on childhood CP trajectories have shown associations with a greater likelihood of school dropout (Bevilacqua et al. 2018; Odgers et al. 2008), increased alcohol and cannabis use during adolescence (Bevilacqua et al. 2018; Kretschmer et al. 2014), as well as lower life satisfaction (Estévez et al. 2018), research examining the consequences associated with IA trajectories has focused primarily on delinquent behaviors and internalizing symptoms. Thus, the present study extends existing knowledge by examining IA trajectories in relation to a broader range of outcomes than has previously been considered.

The aim of this study is to determine whether frequent and persistent IA use from childhood to early adolescence is associated with adjustment difficulties in late adolescence, above and beyond CP. In a previous study examining predictors of IA, four IA trajectories were identified, within the same sample, including one characterized by high and persistent IA use (Boutin et al. 2023). The present study compares these four trajectories on multiple indicators of later adjustment across the individual (depressive and anxiety symptoms, life satisfaction, substance use, delinquent behaviors), social (social support), and academic (school dropout risk) domains. We hypothesize that children belonging to the high and persistent IA trajectory will exhibit greater adjustment difficulties in late adolescence than children in the low IA trajectory. A second objective is to determine whether these differences remain after controlling for CP. Although limited, prior studies controlling for physical aggression suggest that they will.

## 4 Methods

### 4.1 Participants

The aim of the longitudinal research underlying the present study was to gain a better understanding of early CP in girls and boys. Participants (*N* = 744) were recruited between 2008 and 2010 from 155 French‐speaking elementary schools in four administrative regions of Quebec, Canada. To recruit a large sample of children with CP, two strategies were deployed. The first was to invite all girls under 10 years of age, and approximately one in four randomly selected boys, receiving complementary educational services because of their CP to take part in the study (participation rate = 75.1%). Children were assessed by their parents and teachers using the Diagnostic and Statistical Manual of Mental Disorders (DSM) criteria‐oriented scales for oppositional and conduct problems from the Achenbach System of Empirically Based Assessment (ASEBA; Achenbach and Rescorla 2001). Students scoring at or above the 93rd percentile (ASEBA risk and clinical areas) based on parent or teacher report were considered to have CP (*n* = 339). Girls were oversampled in this first strategy because it is well documented that boys are more frequently targeted than girls to receive complementary educational services for CP (see Verlaan et al. 2018).

The second strategy used a systematic classroom screening of 881 students in first to third grade (participation rate = 71.5%). This complementary strategy was chosen to identify children who present CP symptomatology, but who were not signaled to school professionals by teachers and thus were not receiving educational services because of their CP. Parents and teachers rated the same previously mentioned ASEBA scales. An additional 95 children (57.9% girls) had a score above the set threshold and were included in the study. In total, 434 children with CP (44.7% girls) were recruited. For comparison purposes, students who had scored below the 93rd percentile were randomly selected from the systematic classroom screening (*n* = 310; 49.7% girls).

Upon entering the study, students were 8.4 years old on average (age range = 6.6–10.3; SD = 0.93) and 93% were born in Quebec (Canada). Half came from intact families, 20% from blended families and 30% from single‐parent families. Median annual family income ranged from $50,000 to $59,999 CAD, which was below the median annual income of Canadian families ($69,860 CAD) according to 2010 census data (Statistics Canada 2016). More than two thirds of children attended a school in high poverty neighborhoods. No differences emerged in rates of participation of boys and girls, grade level, or poverty level of the school attended (Government of Quebec 2013b).

The design of the longitudinal study involved repeated measures every 12 months. IA trajectories were measured using the first six data collections (T1 to T6). The participation rate varied from one measurement time to the next but amounted to 89.2% at T6 with 664 participants (46.4% girls; mean age = 13.2 years [age range = 11.2–15.8]; SD = 0.95). Outcomes were measured at the end of adolescence at the 10th data collection (T10). The participation rate was still very high at 87.5%, with 651 participants (45.9% girls) at an average age of 17.4 years (age range = 15.3–20.1; SD = 0.98).

### 4.2 Measures

#### 4.2.1 IA Behaviors (T1 to T6)

IA behaviors were assessed by both the parent and teacher using 10 items (e.g., ‘Tells bad of false stories about others’) from the *Direct and Indirect Aggression Scales* (Björkqvist et al. 1992b). They were measured on a 5‐point Likert scale ranging from 0 (*never*) to 4 (*very often*). Given the more subtle nature of IA, these behaviors may be more difficult to observe. Thus, a composite score was computed for each statement, where the highest score between parent and teacher was retained. An average scale score was then computed. A higher score means more frequent use of IA by the student. The internal consistency of the instrument was excellent (Cronbach's alpha ranging from 0.92 to 0.94).

#### 4.2.2 Adjustment Difficulties Assessed by the Participants at T10

##### 4.2.2.1 Symptoms of Depression and Anxiety

Depressive symptoms (13 items; *α* = 0.81; e.g. ‘There is very little that I enjoy’) and anxiety symptoms (six items; *α* = 0.72; e.g. ‘I am nervous or tense’) were measured using the DSM‐criteria oriented scales for depressive and anxiety problems of the *ASEBA Youth Self‐Report* (Achenbach and Rescorla 2001). A sum of the responses to the items, evaluated on a 3‐point Likert scale ranging from 0 (*not true*) to 2 (*very true or often true*), was computed and converted into a T‐score according to ASEBA standards. A high score indicates a high level of depressive and/or anxiety symptoms.

##### 4.2.2.2 Life Satisfaction

Participants were asked to indicate the extent to which they agreed or disagreed with five items (*α* = 0.90) of the *Satisfaction with Life Scale* (Diener et al. 1985), which measures individuals‘ judgment of their life (e.g., ‘In most ways my life is close to my ideal’). The scale is in 7 points, ranging from 1 (*strongly disagree*) to 7 (*strongly agree*). Responses to the items were added up: the higher the score, the more satisfied participants were with their life.

##### 4.2.2.3 Substance Use

The DEP‐ADO (Landry et al. 2005), a screening grid for adolescents‘ alcohol and drug use, was used. It measures the frequency of alcohol, cannabis and other drug consumption, as well as the consequences associated with use (e.g., ‘You had trouble at school because of your alcohol or drug use’). A total score was calculated (*α* = 0.85), ranging from 0 to 100. The higher the score, the more problematic the participant's substance use.

##### 4.2.2.4 Delinquent Behaviors

Fourteen items (*α* = 0.77) from the criminal delinquency scale of the *Measures of social and personal adjustment for Quebec adolescents* (LeBlanc 1996) were used to measure delinquent behaviors. This scale describes behaviors involving physical aggression (e.g., ‘Getting into a fist fight with another person’), vandalism (e.g., ‘Breaking or destroying things that don't belong to you on purpose’) and theft (e.g., ‘Breaking down a door or window and breaking in to get something’). Participants were asked to answer yes if they ever done the behavior described at least once in their life. A sum of the behaviors displayed was then calculated, and the higher this sum, the more delinquent behaviors the participants had engaged in.

##### 4.2.2.5 Social Support

Using the *Social Support Network Map* (Desmarais et al. 1982), participants were asked to identify the people who make up their social network, that is, people with whom they have a relationship, or people they know can provide services or help when needed. These people could be friends, close or distant family members, teachers or coaches, or caregivers. A sum of the number of people named was calculated. The lower the sum, the more socially isolated is the participant.

##### 4.2.2.6 School Dropout Risk

Seven items from the *Potential Dropout Assessment Toolkit* (Janosz et al. 2007) were used to calculate participants' probability of dropping out: academic performance (measured by average grades in language and math), number of grade repetitions and school engagement (whether the student likes school, the student's ranking with respect to the average, the importance of getting good grades and the desired level of education). The probability of dropping out is a continuous score ranging from 0 to 1, where a higher score indicates a greater risk of dropping out. For participants no longer attending high school at T10, if they dropped out before graduating, their risk was coded as 1, and as 0 if they graduated.

#### 4.2.3 Control Variables

Since the predictors consisted of trajectories measured from T1 to T6, control variables assessed at T6 were retained because they were measured closer in time to the outcomes assessed at T10.

##### 4.2.3.1 Age, Sex and Income

Chronological age at T6 was calculated, considering the date of birth declared by the parents and the date of the survey's completion. Regarding sex, parents indicated whether their child was born as a girl (coded 0) or a boy (coded 1). Family income at T6 was assessed using an adjusted version of the ordinal scale from the *Quebec survey on children's mental health* (Valla et al. 1994). The parent was asked to classify family income in categories ranging from 0 (less than $6000) to 15 (more than $150,000). The variable was weighted to ensure equal distance between each of its units. The average value is 7.13, ranging from $70,000 to $79,999 (all values in CAD).

##### 4.2.3.2 CP

Participants‘ CPs at T6 were measured using the DSM Oriented Scale for Conduct Problems completed by the parent (17 items; *α* = 0.87) and teacher (13 items; *α* = 0.91) of the ASEBA (Achenbach and Rescorla 2001). Statements (e.g., ‘Breaks rules at home, school, or elsewhere’; ‘Threatens people’) were rated on a 3‐point Likert scale ranging from 0 (*not true*) to 2 (*very true or often true*). A sum was calculated and converted into a *T*‐score according to the instrument's standards. The highest *T*‐score between parent and teacher was used. This multi‐respondent approach has been identified as providing an optimal balance between sensitivity and specificity in the assessment of CP, compared to using the score of one of the two informants or the mean score between the two informants (Lapalme et al. 2020).

### 4.3 Procedures

The questionnaires were administered in the form of an at‐home interview conducted by a research assistant. Data on IA and CP were collected from parents, and data on adjustment difficulties from adolescents. Data on delinquent behaviors was collected by self‐completion during the interview. For teachers, data were collected through telephone interviews. A full description of the research and a complete reading of the consent form were carried out for each respondent. Parents had to consent before their child could take part in the research, and before the teacher could be contacted to answer questions about the child's behavior in class. The complete protocol required an average of 90 min for the parent and adolescent, and 30 min for the teacher. Compensation was offered to each respondent. The longitudinal research was approved by the university's ethics committee.

### 4.4 Statistical Analyses

All analyses for this study were performed using Mplus software version 8.10 (Muthén and Muthén 2023). This software handles missing data using Full information maximum likelihood (FIML), a built‐in procedure that does not impute missing values and allows all participants with available date to contribute to the estimated model. In the present study, the proportion of missing data ranged from 0% to 15.9%, and the data were not missing completely at random (Little's MCAR test: *χ^2^* (412) = 525.83, *p* < 0.001). Missingness was primarily attributable to participant attrition over time and to missing data on the school dropout risk score, which was not computed for participants enrolled in vocational training or adult education programs (*n* = 63). Of the 651 participants with data on T10, 640 also had data available for at least three time points between T1 and T6. These 640 participants were compared with the 104 participants who had missing data at T10 and missing data for more than three time points between T1 and T6. No statistically significant differences were found between the two groups in terms of age at T1, *t*(742) = −1.51, *p* = 0.13, family income at T1, *t*(734) = 0.738, *p* = 0.46, or sex, *χ* ^2^(1) = 1.29, *p* = 0.26. Participants with missing data were also not more likely to belong to a specific IA trajectory, *χ* ^2^(3) = 4.03, *p* = 0.26, or to present CP at study inception, *χ* ^2^(1) = 0.86, *p* = 0.35. Furthermore, when missing data were examined within the subsample of 640 participants and the school dropout risk variable was excluded, the proportion of missing data was minimal (< 5%, except for family income, 8.3%), and Little's MCAR was non‐significant, *χ* ^2^(214) = 244.82, *p* = 0.07. These results suggest that, aside from participant attrition and missingness related to the school dropout risk score, the remaining missing data were missing completely at random.

**Figure 1:**
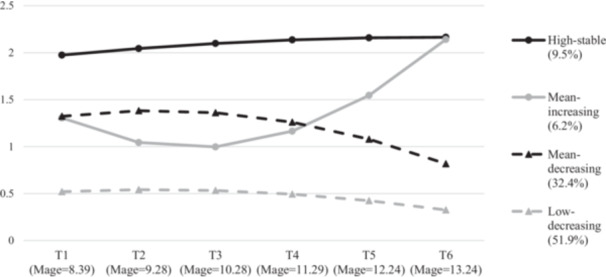
Trajectories of IA use from T1 to T6. *Note*: From Boutin et al. 2023. Copyright 2023 by the American Psychological Association. Reprinted with permission.

To examine whether frequent and persistent IA use in childhood is associated with adjustment difficulties in late adolescence, the first step was to identify trajectories of IA use. As mentioned, a latent class growth model was fitted in a previous study (see Boutin et al. 2023 for statistical details) and the trajectories are replicated here (see Figure 1). Four trajectories were identified from T1 (mean age = 8.4) to T6 (mean age = 13.2): a low‐decreasing trajectory (*n* = 386; 44.3% girls), a mean‐decreasing trajectory (*n* = 241; 46.9% girls), a high‐stable trajectory (*n* = 71; 59.2% girls), and a mean‐increasing trajectory (*n* = 46; 47.8% girls). The proportion of boys and girls did not vary significantly across trajectories (*χ* ^2^(3) = 5.34, *p* = 0.15).

The second step was to link these trajectories to future outcomes measured at T10 (mean age = 17.4). This was done using the manual method of Bolck, Croon and Hagenaars (BCH; Bolck et al. 2004). This method is recommended to examine differences in means between groups of individuals (in this case latent trajectories) on distant outcome variables, since it considers the unequal variances of the variables and the inherent measurement error of latent trajectory class analyses (Asparouhov and Muthén 2014; Nylund‐Gibson et al. 2019). These analyses enable the classification of individuals into different trajectories. However, this classification is based on probabilities, and participants are assigned to the trajectory with which they are most compatible (this compatibility is rarely 100%). Thus, the BCH approach considers the individual error rate for each participant. First, these rates are extracted from the latent class trajectory model, and then, their inverse logits are included as training weights in the estimation of auxiliary models (Asparouhov and Muthén 2014; Nylund‐Gibson et al. 2019). To examine outcome differences between the trajectories, each outcome was entered into the model individually, and Wald tests and post hoc analyses were performed. Where significant differences in means emerged, the effect size (Cohen's *d*) was calculated using the following formula: $\frac{M_{1}-M_{2}}{S_{\mathrm{pooled}}}$ where the estimated means are represented by *M* and the standard deviation (mean of the standard deviations of the two trajectories) is represented by *S*. According to Cohen (1988), *d* = 0.20 indicates a small effect size, *d* = 0.50 a moderate effect size, and *d* = 0.80 a large effect size.

## 5 Results

### 5.1 IA and Future Adjustment Difficulties, Without Considering CP

Descriptives statistics for IA, CP, and adjustment difficulties are presented in Table 1. Because anxiety symptoms did not significantly differ across IA trajectories in the uncontrolled model, this outcome was not considered in subsequent analyses. First, mean levels of adjustment difficulties were compared across IA trajectories while accounting for control variables (age, sex, and income) but not CP. Results indicate significant mean differences between the low‐decreasing trajectory and the three other trajectories (see Table 2). Participants who used low levels of IA over childhood show fewer adjustment difficulties: they engage in less problematic substance use, display fewer delinquent behaviors, and face a lower risk of school dropout. They also express greater life satisfaction and fewer depressive symptoms than children following a high‐stable or a mean‐decreasing trajectory. Moreover, children in the high‐stable trajectory report higher levels of substance use, delinquent behaviors, and school dropout risk than those in the mean‐decreasing trajectory. As for social support, trajectory means were not different, with the Wald test reaching only marginal significance. Finally, no significant difference emerged between the high‐stable and mean‐increasing trajectories, and the only significant difference between the mean‐increasing and mean‐decreasing trajectories is that children following a mean‐increasing trajectory show a higher risk of school dropout.

**Table 1:** Descriptives statistics for the variables under study.

Variable	N	Mean (SD)	Minimum	Maximum
IA T1	744	0.98 (0.76)	0	3.6
IA T2	714	0.98 (0.73)	0	4
IA T3	706	0.99 (0.72)	0	4
IA T4	691	0.96 (0.78)	0	4
IA T5	687	0.88 (0.77)	0	3.8
IA T6	664	0.79 (0.79)	0	3.8
CP T6	664	60.19 (8.78)	50	98
Depressive symptoms T10	632	56.39 (7.64)	50	90
Anxiety symptoms T10	632	55.05 (6.65)	50	80
Life satisfaction T10	632	26.33 (6.77)	5	35
Substance use T10	631	37.85 (24.50)	10	100
Delinquent behaviors T10	632	1.26 (1.91)	0	13
Social support T10	632	8.87 (4.68)	0	38
School dropout risk T10	580	0.40 (0.40)	0.00	1

**Table 2:** Adjustment difficulties mean differences between IA use trajectories, without considering CP.

		Controls			Trajectories means						
Outcome variables	*n*	Age (*p* value)	Sex (*p* value)	Income (*p* value)	LD estimated mean (SD)	MD estimated mean (SD)	MI estimated mean (SD)	HS estimated mean (SD)	Wald test (*p* value)	Significant post hoc comparisons (Cohen's *d*)	
Depressive symptoms	572	0.31 (0.35)	−1.36 (0.04)	−0.04 (0.66)	55.25 (7.62)	57.51 (7.62)	56.67 (7.62)	58.16 (7.62)	8.00 (0.046)	HS > LD	MD > LD
										(0.38)	(0.30)
Life satisfaction	572	−0.48 (0.08)	0.65 (0.25)	0.16 (0.01)	27.57 (6.61)	25.44 (6.62)	24.87 (6.60)	23.86 (6.62)	11.69 (0.01)	HS > LD	MD > LD
										(0.56)	(0.32)
Substance use	571	6.25 (0.00)	6.95 (0.00)	0.40 (0.06)	31.78 (23.15)	40.57 (23.07)	44.02 (22.72)	54.28 (22.82)	50.03 (0.00)	HS > LD	MD > LD
										(0.98	(0.38)
										MI > LD	HS > MD
										(0.53)	(0.60)
Delinquent behaviors	572	0.22 (0.01)	0.85 (0.00)	−0.01 (0.57)	0.74 (1.80)	1.60 (1.80)	1.76 (1.78)	2.36 (1.78)	42.80 (0.00)	HS > LD	MD > LD
										(0.91)	(0.48)
										MI > LD	HS > MD
										(0.57)	(0.42)
Social support	572	−0.28 (0.21)	0.35 (0.36)	0.18 (0.00)	9.74 (4.65)	8.41 (4.66)	7.85 (4.65)	7.83 (4.66)	6.96 (0.07)		
School dropout risk	529	−0.05 (0.00)	0.10 (0.00)	−0.03 (0.00)	0.21 (0.34)	0.50 (0.34)	0.73 (0.35)	0.79 (0.33)	120.37 (0.00)	HS > LD	MD > LD
										(1.73)	(0.85)
										MI > LD	
										(1.51)	
										HS > MD	MI > MD
										(0.87)	(0.67)

Abbreviations: HS, high‐stable trajectory; LD, low‐decreasing trajectory; MD, mean‐decreasing trajectory; MI, Mean‐increasing trajectory.

### 5.2 IA and Future Adjustment Difficulties, Considering CP

**Figure 2:**
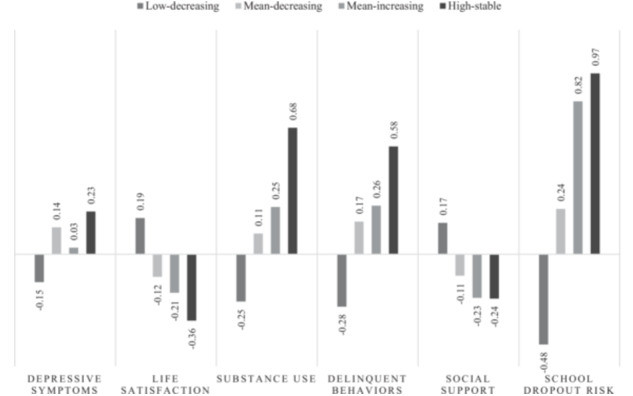
Adjustment difficulties mean differences between IA use trajectories. *Note:* Mean values were standardized to allow comparisons across outcomes.

In the final set of analyses, mean levels of adjustment difficulties were compared across trajectories while accounting for both control variables and CP (see Table 3). CP at the beginning of high school (T6) predict most of adjustment difficulties, except for substance use and social support. Mean differences in depressive symptoms and life satisfaction between IA trajectories were no longer significant. However, trajectories continued to differ on substance use, delinquent behaviors, and school dropout risk. Specifically, children in high‐stable and mean‐decreasing trajectories still reported greater adjustment difficulties than those in the low‐decreasing trajectory after controlling for CP. Additionally, children in the high‐stable trajectory still reported higher substance use than those in the mean‐decreasing trajectory, and children in the mean‐increasing trajectory continued to face a higher risk of school dropout than those in the mean‐decreasing trajectory. In contrast, differences between the mean‐increasing and low‐decreasing trajectories were no longer significant. Regarding effect sizes, half exceeded 0.80, indicating large effects, whereas the smallest effect size was 0.34, observed for the comparison of substance use between the mean‐decreasing and low‐decreasing trajectories. Outcome differences across trajectories are illustrated in Figure 2.

**Table 3:** Adjustment difficulties mean differences between IA use trajectories, considering CP.

		Controls				Trajectories means						
Outcome variables	*n*	Age (*p* value)	Sex (*p* value)	Income (*p* value)	CP (*p* value)	LD estimated mean (SD)	MD estimated mean (SD)	MI estimated mean (SD)	HS estimated mean (SD)	Wald test (*p* value)	Significant post hoc comparisons (Cohen's *d*)	
Depressive symptoms	572	0.38 (0.25)	−1.51 (0.02)	0.002 (0.98)	0.18 (0.01)	55.25 (7.52)	57.51 (9.95)	56.67 (9.72)	58.16 (8.52)	2.94 (0.40)		
Life satisfaction	572	−0.57 (0.04)	0.85 (0.13)	0.114 (0.09)	−0.232 (0.00)	27.57 (6.67)	25.44 (8.08)	24.87 (9.30)	23.86 (7.67)	1.94 (0.58)		
Substance use	571	6.37 (0.00)	6.69 (0.00)	0.47 (0.03)	0.31 (0.13)	31.78 (22.15)	40.57 (29.19)	44.02 (30.86)	54.28 (29.38)	13.05 (0.01)	HS > LD	MD > LD
											(0.87)	(0.34)
											HS > MD	
											(0.47)	
Delinquent behaviors	572	0.23 (0.00)	0.81 (0.00)	−0.002 (0.91)	0.037 (0.03)	0.74 (1.43)	1.60 (2.50)	1.76 (2.45)	2.36 (2.62)	8.84 (0.03)	HS > LD	MD > LD
											(0.80)	(0.44)
Social support	572	−0.29 (0.18)	0.40 (0.30)	0.17 (0.00)	−0.05 (0.21)	9.74 (5.36)	8.41 (5.42)	7.85 (3.95)	7.83 (4.04)	1.70 (0.64)		
School dropout risk	529	−0.05 (0.00)	0.09 (0.00)	−0.03 (0.00)	0.01 (0.00)	0.21 (0.34)	0.50 (0.47)	0.73 (0.40)	0.79 (0.37)	16.91 (0.00)	HS > LD	MD > LD
											(1.63)	(0.72)
											MI > LD	
											(1.41)	

Abbreviations: HS, high‐stable trajectory; LD, low‐decreasing trajectory; MD, mean‐decreasing trajectory; MI, mean‐increasing trajectory.

## 6 Discussion

The present study investigated whether and how trajectories of IA from childhood to adolescence relate to adjustment in late adolescence, while accounting for childhood CP. Overall, our findings highlight that beyond the contribution of childhood CP, distinct IA trajectories are uniquely associated with later maladjustment, with the high‐stable trajectory remaining associated with problematic substance use, delinquent behaviors, and risk of school dropout.

### 6.1 IA and Future Adjustment Difficulties

Regarding internalizing symptoms, our results indicate that children in the mean‐decreasing and high‐stable trajectories reported higher levels of depressive symptoms than those in the low‐decreasing trajectory. However, this difference became non‐significant after controlling for CP. Further, no significant differences emerged for anxiety. Previous studies examining the association between IA trajectories and internalizing symptoms have often measured these symptoms broadly or relied on depression‐specific scales, and they generally also report non‐significant associations (Cleverley et al. 2012; Ehrenreich et al. 2016; Underwood et al. 2011). One possible explanation is that IA may be more strongly related to externalizing difficulties, such as delinquency or substance use, than to internalizing problems. This interpretation is supported by the meta‐analysis of Card et al. (2008), which found that although IA is significantly associated with internalizing problems (*r* = 0.10), its association with delinquent behaviors is much stronger (*r* = 0.45). Consistently, Tackett et al. (2013) also demonstrated that IA behaviors belong to the externalizing spectrum. Another explanation, supported by our findings, is that children who engage in IA frequently and persistently may also exhibit CP. While IA alone may contribute to depressive symptoms through mechanisms such as interpersonal conflicts and poorer relationship quality (Dryburgh et al. 2022), the stronger link with depression may be driven by their CP behaviors. Behaviors such as defiance toward authority figures or physical aggression may lead to more severe interpersonal, academic, and disciplinary difficulties, which in turn could weigh more heavily on youth's psychological well‐being than IA behaviors alone.

For life satisfaction, the mean‐decreasing and the high‐stable trajectories initially differed from the low‐decreasing trajectory, but these differences were no longer significant once CP were considered. As such, youth engaging in IA across childhood, either at increasing, decreasing or high‐stable levels, do not report lower life satisfaction as a direct consequence of these conducts. Instead, the observed differences seem attributable mostly to CP. Various outcomes associated with childhood CP trajectories may contribute to the lower life satisfaction observed in this study, including an increased likelihood of poor health, limited education, and unemployment (Bevilacqua et al. 2018). As for social support, analyses indicated that income accounted for the variance rather than IA behaviors or CP. Frequently reported in the literature, this association may reflect the constraints imposed by low income, like financial strain, inconsistent work hours and living in deprived neighbourhoods, which all limit the quantity and quality of social relationships (Hjalmarsson and Mood 2015; Kung et al. 2022).

One of the key findings of this study concerns differences in adolescent externalizing behaviors across IA trajectories. Even after controlling for CP, youth in the mean‐decreasing and high‐stable IA trajectories reported more problematic substance use, greater delinquent behaviors, and an increased risk of school dropout than those in the low IA trajectory. These results align with a growing body of research demonstrating consistent differences in rule‐breaking, delinquency, and risk‐taking behaviors between IA trajectories measured through childhood and/or adolescence (Chen et al. 2019; Cleverley et al. 2012; Ehrenreich et al. 2016; Spieker et al. 2012; Underwood et al. 2011). A potential explanation for these results lies in the role of popularity. Research consistently shows that adolescents who engage in IA are perceived as popular (see Hensums et al. 2023 for a meta‐analysis). Popular youth benefit from advantages not available to their less popular peers, such as access to exclusive social opportunities, including unsupervised parties (Laursen and Veenstra 2021) where substance use is more likely to occur (Lee and Vandell 2015). In addition, popularity is often associated with the adoption of at‐risk or “adult” behaviors (i.e., behaviors unauthorized to underage individuals) that signal autonomy from adults and are valued by peers (Schwartz and Gorman 2011). These behaviors include alcohol and drug use, delinquency, and disengagement from school. In turn, being defiant, or less engaged in school, and adopting at‐risk behaviors affect school dropout risk (Hawkins et al. 2013). Thus, a high and stable use of IA may constitute a risk factor for later adjustment difficulties through its association with elevated social status and the behavioral patterns linked to popularity. Popularity may therefore represent a key mechanism underlying the pathway from IA to future adjustment difficulties, an interpretation that warrants further investigation in future studies.

Associations involving control variables were generally consistent with previous literature (e.g., Barbot and Hunter 2012; Chen and Jacobson 2012; Goldbeck et al. 2007; Gubbels et al. 2019; Martin 2019; McFarland et al. 2019; Salk et al. 2017). Older age was associated with lower life satisfaction, greater problematic substance use, and more delinquent behaviors, while also being linked to a lower risk of school dropout, likely reflecting the coding of this variable whereby students who had graduated from high school received a score of 0. Higher income was associated with greater social support and a lower risk of school dropout, but also with greater problematic substance use. Finally, boys reported greater problematic substance use and delinquent behaviors, a higher risk of school dropout, and fewer depressive symptoms.

## 7 Limits, Strengths and Conclusion

The findings of this study should be interpreted considering certain limitations. First, from late childhood onward, parent and teacher reports may become less reliable for assessing IA, as these behaviors tend to become increasingly subtle and difficult for adults to detect at this developmental stage (Underwood et al. 2018). Nevertheless, the use of two independent informants helps to somewhat mitigate this limitation. Second, our analyses relied on a single assessment of CP, whereas modeling CP trajectories might have revealed different, and potentially more complex, patterns of results. This represents an avenue for future research. Third, because children with elevated levels of CP were oversampled in this study, the findings may have limited generalizability to the broader population, where such elevated levels are less common.

One of the strengths of this study lies in the use of IA trajectories from childhood through adolescence, in contrast to a single assessment of IA. This approach makes it possible to identify children who engage in IA at high and persistent levels, thus going beyond occasional use that may be considered normative in childhood. In addition, the use of the BCH method constitutes another strength, as this approach does not assume perfect classification of participants into trajectories and accounts for imprecision in group assignment. Finally, the outcomes were measured 4 years after the trajectories, thereby providing evidence of long‐term consequences.

Consistent with expectations, our findings indicate that although CP are a robust predictor of adjustment difficulties in adolescence, IA behaviors are also linked to specific outcomes with non‐negligible effect sizes. They also suggest that IA behaviors are embedded within the broader behavioral profile of children with CP as shown in previous studies (e.g., Ackermann et al. 2019; Boutin et al. 2021). A mean or high IA trajectory may thus represent a risk factor in itself, yet these behaviors often co‐occur with CP, complicating the interpretation of their unique contribution and underscoring the need for more studies examining adjustment difficulties associated with IA while accounting for CP.

While children with CP often receive psychosocial services, IA is rarely the focus of such interventions. Indeed, these behaviors are often overlooked in school assessments (Loeber et al. 2009), and most prevention programs primarily target direct forms of aggression (Shen et al. 2025). By focusing on overt CP manifestations (e.g., physical or verbal aggression), interventions may even inadvertently encourage more subtle behaviors such as IA. Given that victims often perceive IA as more distressing than direct aggression (i.e., they report higher internalizing problems; Casper and Card 2017) and that these behaviors undermine peer relationships, prevention and intervention efforts must explicitly target IA alongside CP. Importantly, because IA frequently goes unnoticed and youth who engage in it may be less likely to seek help, schools play a central role in early detection. Providing school staff with tools to recognize and respond to IA is therefore crucial to ensure timely and effective intervention, as less visible behaviors can still drive high‐impact risks.

## Data Availability

The data that support the findings of this study are available on request from the corresponding author. The data are not publicly available due to privacy or ethical restrictions.

## Footnotes

1 The terms relational aggression and social aggression are also used in the literature to describe similar behaviors. Although nuanced, all three refer to aggression targeting a victim's social status and peer acceptance (Murray‐Close et al. 2016). In line with our methodology, we use the term indirect aggression, while considering studies employing any of these concepts
